# TNFα induces co-trafficking of TRPV1/TRPA1 in VAMP1-containing vesicles to the plasmalemma via Munc18–1/syntaxin1/SNAP-25 mediated fusion

**DOI:** 10.1038/srep21226

**Published:** 2016-02-18

**Authors:** Jianghui Meng, Jiafu Wang, Martin Steinhoff, James Oliver Dolly

**Affiliations:** 1 International Centre for Neurotherapeutics, Dublin City University, Glasnevin, Dublin 9, Ireland; 2 Department of Dermatology and Charles Institute for Translational Dermatology, University College Dublin, Ireland

## Abstract

Transient receptor potential (TRP) A1 and V1 channels relay sensory signals, yet little is known about their transport to the plasmalemma during inflammation. Herein, TRPA1 and TRPV1 were found on vesicles containing calcitonin gene-related peptide (CGRP), accumulated at sites of exo- and endo-cytosis, and co-localised on fibres and cell bodies of cultured sensory neurons expressing both. A proinflammatory cytokine, TNFα, elevated their surface content, and both resided in close proximity, indicating co-trafficking. Syntaxin 1–interacting protein, Munc18–1, proved necessary for the response to TNFα, and for TRPV1-triggered CGRP release. TNFα-induced surface trafficking of TRPV1 and TRPA1 required a synaptic vesicle membrane protein VAMP1 (but not 2/3), which is essential for CGRP exocytosis from large dense-core vesicles. Inactivation of two proteins on the presynaptic plasma membrane, syntaxin-1 or SNAP-25, by botulinum neurotoxin (BoNT)/C1 or /A inhibited the TNFα-elevated delivery. Accordingly, enhancement by TNFα of Ca^2+^ influx through the upregulated surface-expressed TRPV1 and TRPA1 channels was abolished by BoNT/A. Thus, in addition, the neurotoxins’ known inhibition of the release of pain transmitters, their therapeutic potential is augmented by lowering the exocytotic delivery of transducing channels and the resultant hyper-sensitisation in inflammation.

Considerable research has focused on the vanilloid (V1) and ankyrin (A1) varieties of transient receptor potential (TRP) cation channels in sensory neurons, because of their pivotal roles in the transduction of pain signals^1^. TRPV1 detects heat (>43 °C), acid and various chemicals including capsaicin^2^,^3^. The presence of at least 6 ankyrin repeats at its N-terminus is essential for channel functioning, as well as numerous protein-protein interactions. On the other hand, TRPA1 which has 14–18 ankyrin repeats is activated by various pungent chemicals or cold temperature (<15 °C)^1^. The two types of TRP channels are expressed on distinct, but overlapping, populations of sensory nerves, thereby, endowing afferent fibres with a complex array of polymodal nociceptive capabilities. In neurons of mouse dorsal root ganglia (DRG), TRPV1 is localised on ~12% of the total population and ~30% of this fraction also contain TRPA1^4^. The latter occurs in <4% of mouse DRG neurons, 97% of which is co-expressed with CGRP and TRPV1^4^. In contrast, a higher population of trigeminal ganglion neurons (TGNs) from neonatal rats express TRPA1 (~20%) and TRPV1 (30–50%)^5^. Activities of both channels are upregulated during chronic pain and itch, after undergoing phosphorylation by increased activity of certain kinases, such as protein kinase A or C, phosphatidylinositol 3-kinase and/or sarcoma kinase^6^,^7^,^8^,^9^,^10^,^11^,^12^,^13^,^14^,^15^,^16^,^17^,^18^. Plasma membrane insertion of TRPV1 is enhanced by inflammatory agents such as nerve growth factor (NGF), bradykinin, insulin and insulin-like growth factor l, but not the cytokine IL-1β^6^,^9^,^10^,^19^,^20^. Also, synaptosomal-associated protein of Mr 25 k (SNAP-25) contributes to the surface expression of TRPV1 in oocytes^12^,^19^, but it remained unknown if SNAP-25 participates in the delivery of TRPA1 to the plasma membrane of sensory neurons. Vesicle-associated membrane protein (VAMP) has been implicated in the later, but the pertinent isoforms have not been determined^18^.

Pro-algesic cytokines, such as tumour necrosis factor-alpha (TNFα), are released from mast cells, lymphocytes, macrophages as well as skin keratinocytes^21^,^22^,^23^. It stimulates the expression and release of calcitonin gene-related peptide (CGRP) and substance P (SP) from sensory and sympathetic neurons^24^,^25^. The TNFα-induced up-regulation of CGRP synthesis involves mitogen-activated protein kinase (MAPK) and p38 pathway^24^. Such released neuropeptides exert a vasodilatory action on vascular smooth muscle by activating their respective receptors. TNFα participates in the genesis of inflammatory and neuropathic pain^26^,^27^, and elicits long-term mechanical allodynia in both naïve and nerve injury models^26^,^27^,^28^. Also, exposure to TNFα elevates sensitivity to capsaicin (i.e. induces high frequency of miniature excitatory spontaneous currents) of superficial dorsal horn neurons in acute slices^29^, and increases TRPV1 sensitivity to heat and capsaicin in mouse DRGs^30^,^31^,^32^. Accordingly, TNF receptor 1 (TNFR1) resides in ~40% of mouse TRPV1-positive DRGs^33^; also, the proportion of TRPV1-expressing DRG neurons is increased by a TNFα-induced TNFR1-response, which involves extracellular signal-regulated kinases^34^. On the other hand, activation of peripheral TRPA1 plays a critical role in the development of TNFα-induced mechanical hyperalgesia, and in sustaining the mechanical hyperalgesia observed after intra-articular injection of complete Freund’s adjuvant to induce arthritis-like symptoms in mice^35^. These data suggest that negating TRPV1 and TRPA1 may reduce neuropathic and arthritic pain. Such an important goal could be achieved using botulinum neurotoxins (BoNTs) that proteolytically inactivate different SNAREs, but their involvement in the exocytotic surface delivery of each channel must first be demonstrated convincingly. Moreover, in relation to the effects of TNFα on TRPV1 and TRPA1, its influence on the channels’ trafficking needs to be established in sensory neurons. Thus, questions to be answered include: (i) does TNFα induce co-trafficking of these TRP channels to the cell surface of sensory neurons; (ii) if so, is this via small clear synaptic vesicles (SCSVs) and/or large dense-core vesicles (LDCVs) and (iii) which precise SNAREs and isoforms are involved.

In this study on rat cultured TGNs, co-localisation of TRP channels with various SNARE proteins and markers for SCSVs and LDCVs was investigated, by dual labelling with their specific antibodies. Extracellular labelling of TRPA1 and TRPV1 during stimulation unveiled their appearance at sites of exo-/endo-cytosis. Lentivirus-mediated expression of fluorescent-tagged TRPV1 and immuno-labelling of endogenous TRPA1 revealed their trafficking, as well as enhanced surface delivery after exposure to TNFα. Ca^2+^ -imaging analysis using agonists selective for TRPV1 or TRPA1 provided functional readout for the augmented surface channels. Expressing TRPV1-V5 tagged fusion facilitated a proximity ligation assay (PLA), which demonstrated interaction and co-trafficking of this channel with TRPA1 upon stimulation with TNFα. The latter seems to involve, at least, a portion of LDCVs that release CGRP from cultured neurons. Moreover, SNARE proteins (SNAP-25, VAMP1 and, possibly, syntaxin1) were found to mediate this trafficking and membrane insertion of TRPV1 and TRPA1, by using serotypes of BoNT that selectively cleave their respective SNAREs^36^. Specific knockdown of Munc18–1, a syntaxin 1-interacting protein, provided the first evidence for its involvement. These new findings should aid the designing of novel therapeutics for normalizing the increased surface appearance of transducing channels and associated neuronal hyper-excitability upon inflammatory/painful stimulation.

## Results

### TRPV1 and TRPA1 co-localize with LDCV-markers and upon depolarization are recruited to exo-/endo-cytotic sites, indicating their transportation via LDCVs

**Figure 1:**
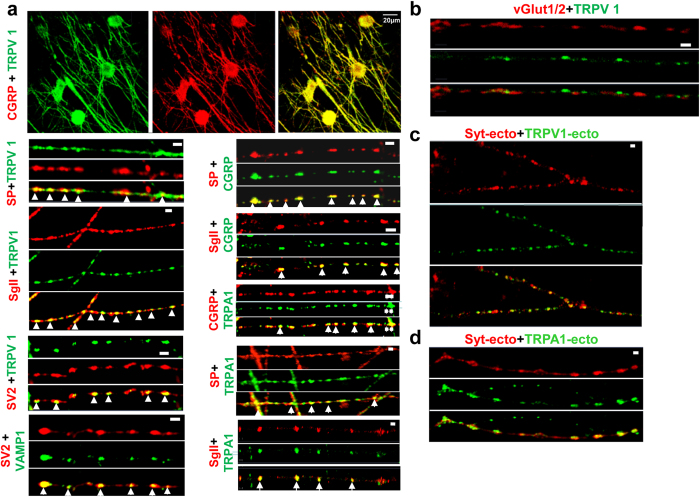
Co-staining TRPV1 or TRPA1 with vesicle markers in rat cultured TGNs: upon depolarization both channels insert at exo-/endo-cytosis sites in the plasmalemma. Permeabilised rat cultured TGNs on 8-well Ibidi chambers were incubated with antibodies for different markers (i.e. neuropeptide and synaptic vesicle proteins). Fluorescently-tagged secondary antibodies were applied: donkey anti-mouse Alexa 594 (1:2000) and donkey anti-rabbit Alexa 488 (1:2000), or donkey anti-goat Alexa 594 (1:2000) and donkey anti-rabbit Alexa 488 (1:2000). (**a**) Notably, a striking co-localisation pattern was visualized upon co-staining with a monoclonal for CGRP (1:1000) and rabbit anti-TRPV1 (1:100), monoclonal SP (1:500) and rabbit anti-TRPV1, goat anti-SgII (1:200) and rabbit anti-TRPV1, SV2 monoclonal (1:50) and rabbit anti-TRPV1, SV2 monoclonal and rabbit anti-VAMP1 (1:1000), monoclonal SP and rabbit anti-CGRP (1:1000), goat anti-SgII and rabbit anti-CGRP, CGRP monoclonal and rabbit anti-TRPA1 (1:100), SP monoclonal and rabbit anti-TRPA1, goat anti-SgII and rabbit anti-TRPA1. Arrows highlight some regions of co-localisation. (**b**) Unlike staining patterns observed in (**a**), TRPV1 did not co-localize with vGlut1/2 (1:500) as revealed by single fibre confocal imaging. (**c**) Live TGNs were incubated with 60 mM [K^+^ ]-depolarisation buffer containing Oyster550-tagged Syt-ecto (1:100) and TRPV1-ecto (1:50) for 10 min before washing and further incubation for 10 min with donkey anti-rabbit Alexa Fluor 488 antibodies. Note that numerous synaptic boutons were stained by both antibodies. (**d**) A similar co-localisation pattern was observed for TRPA1-ecto (1:25) together with Syt-ecto in the live cell labelling experiments. Bars =1 μm, except where indicated. Images shown are representative of 3 independent experiments.

As a prelude to monitoring the channels’ trafficking in sensory neurons, it was pertinent to identify the type of vesicle in which they reside. Rat cultured TGNs were fixed and permeabilized before being incubated with the specific antibodies against TRPV1, SNAREs, secretogranin II (SgII), various neuropeptides, and/or synaptic vesicle protein 2 (SV2). The latter (together with gangliosides) acts as a high-affinity acceptor for a number of BoNT serotypes^37^,^38^,^39^,^40^, and regulates priming of docked vesicles^41^. Using confocal microscopy, TRPV1 was visualized on the cell bodies and co-localized on neurites with LDCV markers (such as SgII, CGRP and SP), as well as SV2 (Fig. 1a); Pearson’s correlation coefficients were 0.93 ± 0.05 for TRPV1&SgII, 0.91 ± 0.02 for TRPV1&CGRP, 0.89 ± 0.03 for TRPV1& SP, 0.9 ± 0.04 for TRPV1&SV2 calculated from 20 images from 3 independent cultures. Counter-staining of SV2 with VAMP1, and CGRP with SP or SgII established that each of the paired antibodies gave similar localisation patterns on the sensory fibres (Fig. 1a). Likewise, TRPA1 displayed striking co-localisation with CGRP, SP and SgII (Fig. 1a), giving Pearson’s correlation coefficients of 0.92 ± 0.03 for TRPA1 and CGRP, 0.89 ± 0.04 for TRPA1 and SP, 0.89 ± 0.03 for TRPA1 and SgII, respectively (calculated from 20 images from 3 independent cultures). In contrast, the distribution of the classic marker of SCSVs [vesicular glutamate transporter 1/2 (vGlut1/2)] very rarely coincided with TRPV1 (Fig. 1b; Pearson’s correlation coefficient = 0.21 ± 0.11, were calculated using 20 images from 3 independent cultures). Such a pronounced occurrence of the channels with neuropeptide-containing LDCVs suggests that they are transported via these vesicles in cultured sensory neurons. To ascertain whether LDCVs containing TRPV1 and TRPA1 can fuse with the plasmalemma in live TGNs, the sensory neurons were labelled with a tagged marker of exo-/endo-cytosis, an antibody (Syt-ecto) against a luminal domain of the synaptic vesicle protein, synaptotagmin I^42^,^43^. This entailed exposing TGNs grown on coverslips to rabbit TRPV1 antibody against an extracellular loop (TRPV1-ecto) and Oyster550-labelled mouse Syt-ecto in 60 mM K^+^ depolarisation buffer for 10 min, before washing and incubation with donkey anti-rabbit Alexa 488 secondary antibody. These experiments unveiled a pronounced occurrence of both signals on the fine fibres (Fig. 1c). The calculated Pearson’s correlation coefficient (0.87 ± 0.12, 20 images from 3 independent cultures) confirmed co-localisation of TRPV1 with Syt-ecto. Notably, a similar outcome resulted from co-staining with TRPA1- and Syt-ecto antibodies (Fig. 1d), giving Pearson’s correlation coefficients of 0.89 ± 0.2, 20 images from 3 independent cultures. These overlapping signals indicate that both TRPV1 and TRPA1 reside near the sites for stimulated exo-/endo-cytosis.

### TNFα upregulates the trafficking of TRPV1 and TRPA1 to the surface of sensory neurons

**Figure 2:**
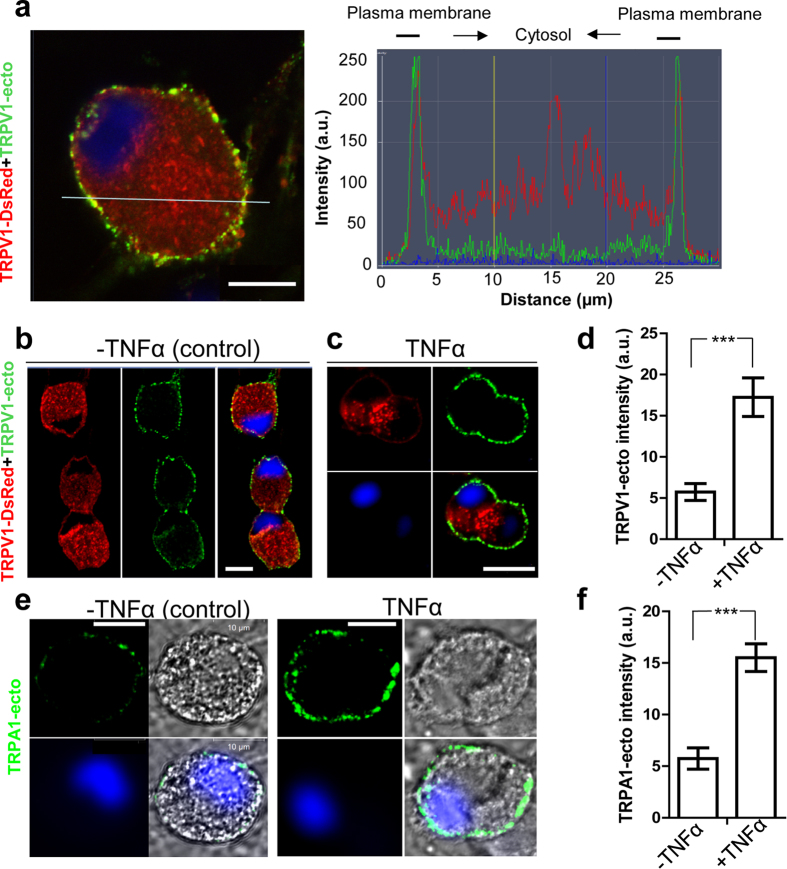
TNFα induces plasmalemma insertion of TRPV1 and TRPA1 in rat TGNs. (**a**) TRPV1-ecto surface labelling of live TGNs infected with lentiviral TRPV1-DsRed was confirmed by confocal microscopy. The exposed TRPV1-DsRed was recognized by TRPV1-ecto antibody (yellow spots on the surface, left panel) under resting condition. Nuclei were counter-stained with DAPI. The red and green fluorescence signals were shown to overlap on the surface area by line scanning analysis (right panel). (**b**) Representative images showing the surface labelling by TRPV1-ecto without TNFα treatment. (**c**) TNFα pre-treatment enhanced the surface binding of TRPV1-ecto over the basal level. Note that lenti TRPV1-DsRed infected TGNs were used in panel **b** and **c**. (**d)** Analysis of >40 images revealed that the fluorescence intensity of TRPV1-ecto was increased ~3 fold by TNFα. (**e**) Representative images, in the fluorescent and phase-contrast modes, of these TGNs showing labelling of surface TRPA1 by TRPA1-ecto, without and with pre-incubation with TNFα. Bars = 10 μm. (**f**) Treatment (24 h) with TNFα caused ~3-fold increment of TRPA1-ecto labelling over the basal level. Data plotted in **d** and **f** are the means ± S.E.M. >40 images analysed from N ≥ 5 independent experiments. The unpaired two-tailed Student’s t-test was used: ***p < 0.001 TNFα V non-treated control.

Cognizant of the report that TNFα increases the number of TRPV1-positive neurons in DRG. (see Introduction), its ability to induce the surface delivery of this protein was examined. TNFα (100 ng/ml) was applied to live TGNs for 24 h, before incubation for 10 min with TRPV1-ecto (in the continued presence of the cytokine) to label the TRPV1 delivered to their surface. After extensive washing and incubation with Alexa 488-conjugated secondary antibody as before, confocal microscope images taken indicated negligible antibody staining of endogenous TRPV1 on the plasmalemma in the absence of TNFα (data not shown). This problem was overcome upon constructing a plasmid by insertion of a gene encoding TRPV1-DsRed fluorescent fusion protein into a lentiviral vector^44^ (Fig. S1a). After infection of TGNs with the resultant lentiviral particles for 7 days, monitoring the total expression of TRPV1-DsRed by SDS-PAGE/Western blotting showed a band with a molecular size ~125 k (Fig. S1b). As expected, the surface expression of TRPV1-DsRed could be readily visualised under the resting condition using the TRPV1-ecto antibody (Fig. 2a, left panel). Analysis by line scanning of the intensities of fluorescence signals for each channel (Fig. 2a, right panel) confirmed that this antibody specifically-labelled external epitopes only. Treatment with TNFα (100 ng/ml) for 24 h, resulted in a significantly (~3 fold) increased TRPV1 labelling on the cell surface over the basal level (Fig. 2b–d).

It was important to investigate whether TRPA1 channels can, also, be up-regulated by TNFα because they were recently found to form hetero-tetrameric complexes with TRPV1 on the plasma membrane of human embryonic kidney cells transfected with their cDNAs^45^, this is proposed to underlie the functional cross-talk involved with activation of sensory neurons in chronic disease conditions^46^,^47^,^48^. Thus, similar experiments were performed by applying TRPA1-ecto antibody to live TGNs and processed (Fig. 2e) as before for TRPV1. Even without over-expression of TRPA1, as needed to detect TRPV1, it could be detected with the TRPA1-ecto antibodies. Compared to the basal, treatment with TNFα induced ~3-fold increment in the fluorescence intensity on the cell surface (Fig. 2f), similar to the increase observed for TRPV1.

### SNAREs in sensory neurons are essential for TNFα-induced membrane insertion of TRPV1 and TRPA1

**Figure 3:**
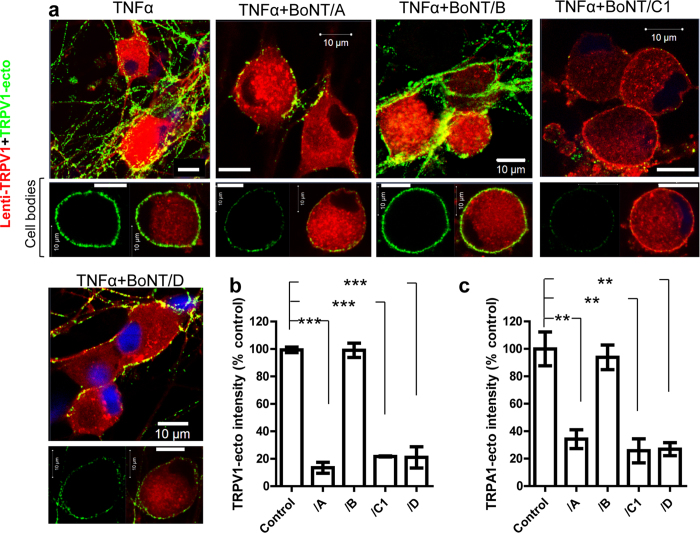
Surface trafficking of TRPV1 and TRPA1 in rat cultured TGNs is blocked by BoNT/A, /C1 or /D but not /B. (**a**) Lenti TRPV1-DsRed infected TGNs were treated with 100 nM BoNT/A, /B, /C1 or /D for 24 h before adding TNFα for 24 h in the presence of toxin and subsequent incubation for 10 min with TNFα, and TRPV1-ecto (green ) for surface labelling. It is apparent from representative images that TNFα enhanced the surface trafficking of TRPV1 and this was reduced by pre-treatment with BoNT/A, C1 or /D but not /B. Bottom images in each panel show the representative labelling of single cell bodies by TRPV1-ecto (left) or merged with TRPV1-DsRed (right). Scale bars = 10 μm. (**b**,**c)** BoNT/A, /C1 and /D significantly blocked membrane insertion of TRPV1 (**b**) and TRPA1 (**c**), revealed using their specific ecto-antibodies; over 40 images from 3 independent experiments were taken for analysis of fluorescent intensity for each treatment and control. Data plotted are means ± S.E.M. One-way ANOVA paired values followed by Bonferroni post hoc test was used: **p < 0.01; ***p < 0.001. Note that there is no significant difference between control and /B treated cells in panel **(b**,**c**).

In terms of possibly finding a therapeutic to attenuate the surface trafficking of pain transducing channels, TRPV1 and TRPA1, we investigated the molecular machinery underlying their TNFα-enhanced transport and insertion into the plasmalemma. Participation of SNAREs in this process was addressed using different BoNTs to truncate and inactivate SNAP-25 (/A, /C1), syntaxin 1 (/C1) or rat VAMP (isoform 1, /D; 2/3, /B, /D)^49^. After infection with lentiviral TRPV1-DsRed, TGNs were incubated with each of the above-noted BoNTs for 24 h at 37 °C followed by the same treatment with TNFα, before application of the TRPV1-ecto antibody for 10 min in the presence of TNFα and processing for SDS-PAGE/Western blotting or microscopy. Notably, BoNT/A, /C1 or /D cleaved ≥80% of their respective SNARE substrates (Fig. S2). Each greatly reduced TRPV1 insertion into the cell surface (Fig. 3a), as demonstrated by calculating the green fluorescence intensity due to surface TRPV1 relative to the BoNT-free control (Fig. 3b). In contrast /B, which truncated >80% of VAMP 2 and 3 but did not cleave VAMP1 (Fig. S2) due to a known mutation of the cleavage site^50^, failed to significantly affect the trafficking of TRPV1 to the plasma membrane (Fig. 3a,b). As BoNT/D additionally cleaved isoform 1 (Fig. S2), our data suggest that VAMP1 rather than VAMP2 or 3 is involved in the surface delivery of TRPV1. This hypothesis is supported by our observation that VAMP1 is important for the exocytosis of neuropeptides, such as CGRP and SP from these sensory neurons^36^. Inhibition of TNFα-induced trafficking of TRPV1 by BoNT/C1 (Fig. 3a,b) could have resulted from its cleavage of syntaxin1 and SNAP-25 (Fig. S2; see Discussion). Similar to that observed for TRPV1, surface labelling of TRPA1 was also significantly decreased by BoNT/A, /C1 or /D, but not /B (Fig. 3c and Fig. S3). The latter is consistent with the notion that VAMP1-containing vesicles mediate membrane insertion of both TRPA1 and TRPV1.

### Direct monitoring of fluorescent VAMPs demonstrated a more prominent location of isoform 1 close to the surface membrane consistent with its exocytotic delivery of TRPV1 and TRPA1

**Figure 4:**
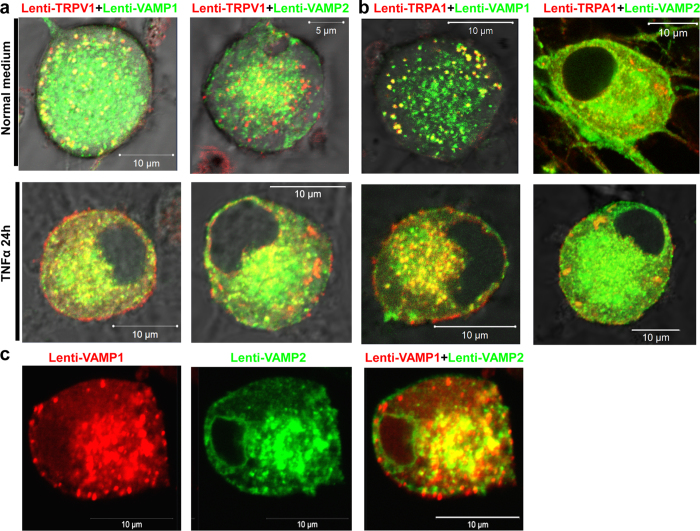
VAMP1, but not isoform 2, is implicated in the membrane insertion of TRPV1 and TRPA1 in rat cultured TGNs: VAMP1 and 2 display distinct intracellular distributions. Cells infected by TRPV1-DsRed together with VAMP1-GFP or VAMP-2-GFP (**a**) or TRPA1-DsRed together with VAMP1-GFP or VAMP-2-GFP (**b**) were treated with or without TNFα (100 ng/ml) for 24 h before fixation and visualization by confocal microscopy. (**c**) Cells were co-infected by lenti VAMP1-DsRed and VAMP2-GFP for 7 days before fixation and visualization by confocal microscopy. Lentiviral-mediated expression of VAMP1-DsRed gave a vesicular-like distribution along the plasma membrane as well as a widely distributed pattern (left), in contrast to VAMP2-GFP which did not yield much labelling along the plasmalemma (middle and right panels). Images shown are representative of 3 independent experiments.

To address why surface delivery of TRPV1 and TRPA1 requires VAMP1 but not 2 or 3 (much less abundant in TGNs, c.f.^49^), lentiviral vector encoding TRPA1-DsRed, VAMP1-GFP, VAMP2-GFP (Fig. S1a) were engineered for directly visualising their cellular distribution and trafficking. After co-infecting TGNs with lentiviruses encoding TRPV1-DsRed with VAMP1-GFP or VAMP2-GFP, expression of these proteins was confirmed by Western blotting, as well as that of the endogenous VAMP isoforms which migrated faster (Fig. S1b). Under the basal condition, TRPV1-DsRed and VAMP1-GFP notably co-localized in granular structures predominantly close to the plasma membrane (Fig. 4a, top left panel); in stark contrast, TRPV1-DsRed and VAMP2-GFP showed mainly distinct intracellular distributions somewhat distant from the plasma membrane, with some overlap in the central region (Fig. 4a, top right panel). After incubating the neurons with TNFα for 24 h, much more TRPV1-DsRed became located on the surface of TGNs (Fig. 4a, bottom left panel) apparently delivered there by VAMP1-GFP containing-vesicles, judging by the presence of the latter near the plasmalemma (Fig. 4a, bottom left panel). In contrast, images of cells producing VAMP2-GFP (Fig. 4a, bottom right panel) display distinct features especially of large clusters of TRPV1-DsRed away from the plasma membrane and much less surface labelling. A similar experiment performed for TRPA1 confirmed that TRPA1-DsRed was also co-localised with VAMP1-GFP in granular structures close to the plasma membrane in TGNs without treatment of TNFα, a pattern distinct from cells infected with TRPA1-DsRed and VAMP2-GFP. TNFα treatment resulted in much more surface TRPA1 in cells expressing VAMP1-GFP than VAMP2-GFP (Fig. 4b). As expected for the lentivirally-expressed non-membranous GFP and DsRed, these displayed uniform distribution throughout the cytoplasm (Fig. S4). Thus, recombinant expression of VAMP1, but not VAMP2, enhanced surface trafficking of TRPV1 and TRPA1. As both VAMP1 and VAMP2 can mediate vesicle fusion and transmitter release from neurons^49^,^51^,^52^, our data raised a very interesting question of whether VAMP1 and 2 should have different distributions. For their direct visualization in live sensory neurons, another lentiviral vector encoding VAMP1-DsRed (Fig. S1a) was generated as before. In TGNs co-transfected with VAMP1-DsRed and VAMP2-GFP lentiviruses, VAMP1-DsRed showed vesicular-like localisation near the plasma membrane, whereas VAMP2-GFP was relatively weak (Fig. 4c, left and middle), and displayed little co-localisation with VAMP1-DsRed along the plasmalemma (Fig. 4c, right). This subcellular distribution of VAMP1 near the plasma membrane distinct from that of isoform 2 is also clearly visible in their individual images (Fig. 4a, top panels), and highlights the importance of VAMP1 in vesicular trafficking of TRP channels in sensory neurons.

### Enhancement by TNFα of Ca^2+^ influx through the upregulated surface-expressed TRPV1 and TRPA1 is normalized by truncation of SNAP-25

**Figure 5:**
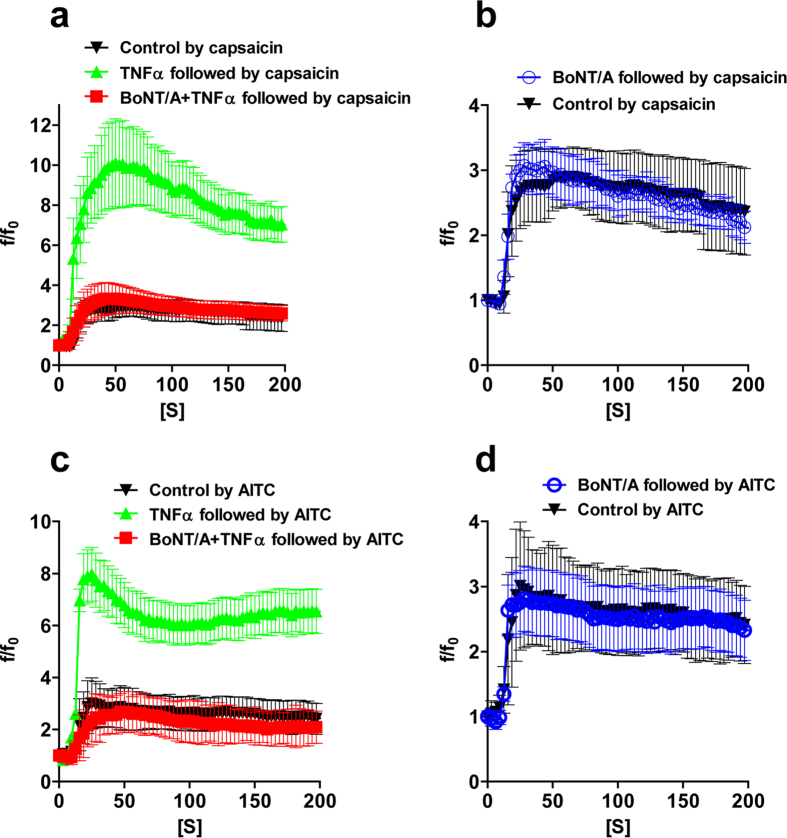
TNFα enhances Ca^2+^ influx in cultured TGNs which is completely blocked by BoNT/A. Rat TGNs, pre-treated with or without 100 nM BoNT/A, were incubated with TNFα for 24 h before measuring capsaicin- or AITC-evoked Ca^2+^ influx with Fluo-4 AM, using confocal microscope imaging. Fluorescent readings (f) at each time point relative to the baseline (f_0_) are plotted. (**a**) Capsaicin-elicited Ca^2+^ -influx in TNFα-treated TGNs was normalized by BoNT/A pre-treatment. (**b**). Capsaicin-elicited Ca^2+^ -influx in control TGNs was unaffected by pre-treatment with BoNT/A. (**c**). TNFα-enhanced AITC-elicited Ca^2+^ -influx was normalized by BoNT/A pre-treatment. (**d**). Ca^2+^ -influx elicited by AITC in control TGNs was not affected by pre-treatment with BoNT/A. Data are the means ± S.E.M; >20 cells recorded from 3 independent culture preparations. Two-way ANOVA with Bonferroni post hoc test analysis for BoNT/A+ TNFα vs control (**a**,**c**), and BoNT/A vs control (**b**,**d**) showed non-significance (p > 0.05) at all the time points; for TNFα vs BoNT/A + TNFα and TNFα vs control (**a**,**c**) gave p < 0.05 at the time points after 25 s.

To determine the functional consequences of blocking the TNFα-induced membrane trafficking of TRPV1 by BoNTs, Ca^2+^ influx through this channel was measured with a selective indicator using capsaicin as the agonist. Cultured TGNs were pre-incubated with Fluo-4-AM in serum-free medium for 20 min before washing and stimulation with 1μM capsaicin^43^. Video recordings were made using a confocal microscope in the time-lapse mode and the average values calculated (Fig. 5a,b). Pre-treatment with TNFα for 24 h significantly increased the Ca^2+^ influx relative to the control, when elicited by capsaicin. In stark contrast, TNFα-enhanced Ca^2+^ influx was normalized to the capsaicin control level by pre-incubation with BoNT/A (Fig. 5a), presumably due to blockade of the increased membrane insertion of TRPV1 normally induced by TNFα. Interestingly, TRPV1-mediated Ca^2+^ influx in TNFα untreated control cells was not affected by BoNT/A (Fig. 5b). In conclusion, TNFα-mediated potentiation of the response of TRPV1 to capsaicin is returned to the non-treated control value by prior exposure to BoNT/A whereas the basal level of Ca^2+^ influx remains unaltered. Similar experiments were performed on TGNs for TRPA1 by measuring Ca^2+^ influx induced with its agonist, allyl isothiocyanate (AITC). Again, pre-treatment with TNFα for 24h significantly raised Ca^2+^ influx in response to 50 μM AITC (Fig. 5c), and this enhancement was reduced by pre-incubation with 100 nM BoNT/A to the level of that for control cells (Fig. 5c). Notably, there was no significant difference between AITC-elicited Ca^2+^ influx in control cells and those pre-treated with BoNT/A (Fig. 5d). These results confirm that TNFα-induced elevation of TRPA1 and TRPV1 activities in mediating Ca^2+^ influx are due to the increased surface delivery of these channel proteins via a SNARE-dependent mechanism.

### Co-trafficking of TRPV1 with TRPA1 visualised directly in sensory neurons was confirmed by their demonstrated proximity in the membrane

**Figure 6:**
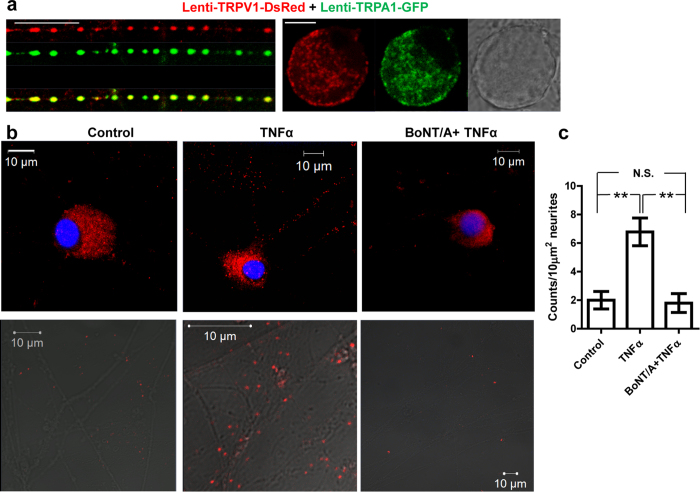
Co-trafficking of TRPV1 and TRPA1 to the plasma membrane in rat TGNs is induced by TNFα and inhibited by BoNT/A pre-treatment. (**a**) Confocal microscopic analysis of the distribution of TRPV1-DsRed and TRPA1-GFP under resting condition on single fibres or cell bodies of rat cultured TGNs infected with lenti TRPV1-DsRed and TRPA1-GFP. (**b**) TGNs infected with lentiviral TRPV1-V5 were treated with or without TNFα for 24 h. PLA was performed following the supplier’s protocol. Note that the red fluorescent spots in the cell bodies and fine neurites shows that TRPV1-V5 resides in proximity to TRPA1 in TGNs. TNFα induced a robust increase in PLA fluorescent signals on the cell bodies and extended neurites which were significantly reduced by BoNT/A to the control level. Bottom row of images show red fluorescent spots on the fibres. Bars in a and b are 10 μm. (**c**) Positive signals for TRPV1-V5 and TRPA1, calculated from the number of red spots in the randomly-selected ROI (10 μm^2^) on the neurites from >40 randomly selected TGNs from 3 independent cultures; this shows that TNFα significantly increased the number of counts, a change prevented by pre-treatment with BoNT/A. Because of the signals being clustered on the cell bodies, it was not possible to quantify these. Data plotted are the means ± S.E.M; One-way ANOVA paired values followed by Bonferroni post hoc test was used: **p < 0.01; N.S., non-significant.

Next, it was pertinent to establish whether TRPV1 and TRPA1 could be transported together with or without TNFα stimulation in the neuronal population that expresses both, using TRPV1-DsRed together with a newly-constructed lentivirus encoding TRPA1-GFP (Fig. S1a). Confocal images of single fibres and cell bodies in TGNs revealed that lentiviral-expressed TRPA1-GFP co-localizes with TRPV1-DsRed (Fig. 6a), supporting the idea that these channels might reside in the same transporting vesicles. This hypothesis was evaluated using a proximity ligation assay (PLA) which is a sensitive measurement of two proteins that reside within <40 nm to each other^53^. PLA required the production of a V5-tagged TRPV1 lentiviral vector as before (Fig. S1a) so its over-expression could afford detection with commercially-available, compatible pairs of antibodies (monoclonal anti-V5 and rabbit antibody against TRPA1). TGNs infected by TRPV1-V5 virus were incubated with or without TNFα for 24 h before performing the PLA. Numerous positive fluorescent spots were detected on cell bodies and extended fibres of the neurons pre-treated with TNFα; in contrast, only a few spots could be observed on the neurites of the control cells (Fig. 6b). Interestingly, BoNT/A treatment of the TGNs diminished the TNFα-induced increase in the number of positive signals down to the level for the control cells (Fig. 6b). Average density of the positive red spots, in regions of interest randomly selected, gave ~7 counts per 10 μm^2^ of fibre for TNFα-treated cells and ~2 for the control; interestingly, pre-treatment with BoNT/A lowered the counts to the control level (Fig. 6b, and analysed in Fig. 6c). It is noteworthy that BoNT/A did not decrease the signals below the control level though the values were at the limit of detection (see Discussion). These collective data provide the first evidence that TRPV1 and TRPA1 are transported together in VAMP1-containing LDCVs to the plasma membrane after stimulation with TNFα, by a process dependent on SNAP-25.

### Munc18–1 is essential for trafficking of TRPV1 and TRPA1 to the cell surface of TGNs

**Figure 7:**
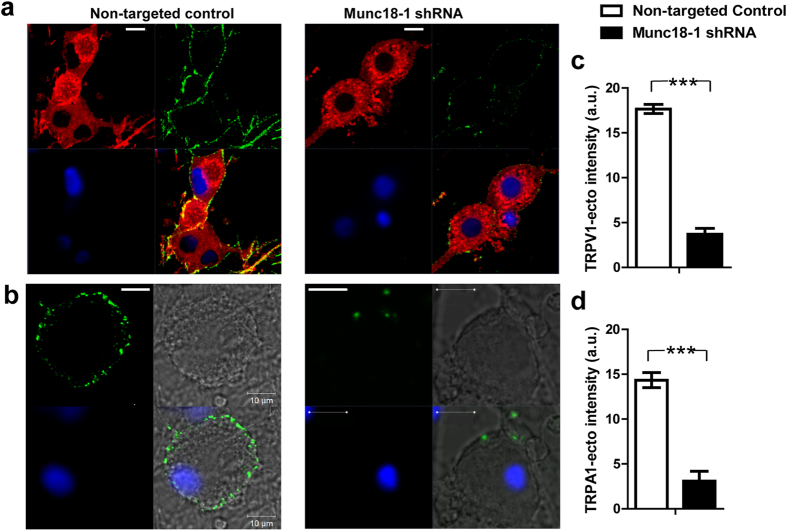
Munc18-1 is required for the trafficking of TRPV1 and TRPA1 to the surface membrane of rat cultured sensory neurons. (**a**) TGNs expressing TRPV1-DsRed were infected with Munc18–1 lentiviral shRNA or its non-targeted control for 7 days. The cells were then incubated with TNFα for 24 h before performing surface labelling of TRPV1. Depletion of Munc18–1 by its specific shRNA significantly reduced the TRPV1-ecto antibody staining, compared to non-targeted control. (**b)** Native TGNs transfected with Munc18–1 lentiviral shRNA or its non-targeted control were incubated with TNFα for 24 h before staining of surface expressed TRPA1. Note that the green fluorescence was lower in the cells infected by Munc18–1 shRNA virus compared to its non-targeted control. Scale bars in (**a**,**b**) are10 μm. Images are representative of 3 independent experiments. The mean fluorescence intensity was quantified for the surface labelled TRPV1 (**c**) and TRPA1 (**d)**. Labelling of both TRPV1 and TRPA1 was reduced by ~80% upon knockdown of Munc18-1. Data plotted are the means ± S.E.M., >20 images from 3 independent experiments. ***<0.001 (the unpaired two-tailed Student’s t-test).

Munc18–1 is required for SNARE complex formation and LDCV release in bovine chromaffin cells^54^ and PC-12 cells^55^. However, a requirement for Munc18–1 in CGRP exocytosis from sensory neurons was not known; likewise, any influence on the trafficking of TRPV1 and TRPA1 remained to be determined. Herein, shRNA-mediated knockdown was used to specifically reduce the expression of Munc18–1. TGNs expressing TRPV1-DsRed were treated for 7 days with lentiviral shRNA specifically targeting Munc18–1 before subjecting the total protein lysate to Western blotting, using Munc18–1 antibody (Fig. S5). In the cells treated with the shRNA, Munc18–1 expression was reduced by 80% ± 1.1 (n = 3) compared to the controls treated with non-targeted shRNA. Importantly, Munc18–1 knockdown resulted in 70% ± 4.3 (n = 3) blockade of capsaicin-stimulated CGRP release compared to the non-targeted control. In separate experiments, after treatment of shRNA for 7 days, cells expressing TRPV1-DsRed were incubated with TNFα for 24 h before application of extracellular antibody against TRPV1. Surface labelling of TRPA1 after shRNA treatment was performed similarly except using TGNs not subjected to infection with lenti TRPV1-DsRed. Those reveal that Munc18–1 knockdown caused ~80% reduction in the content of surface-associated TRPV1 (Fig. 7a,c) and TRPA1 (Fig. 7b,d) labelled with their respective antibodies. Therefore, depletion of Munc18–1 decreases the transportation of both TRPA1 and TRPV1 to the surface of sensory neurons, and this is associated with a reduction of evoked CGRP release.

## Discussion

**Figure 8:**
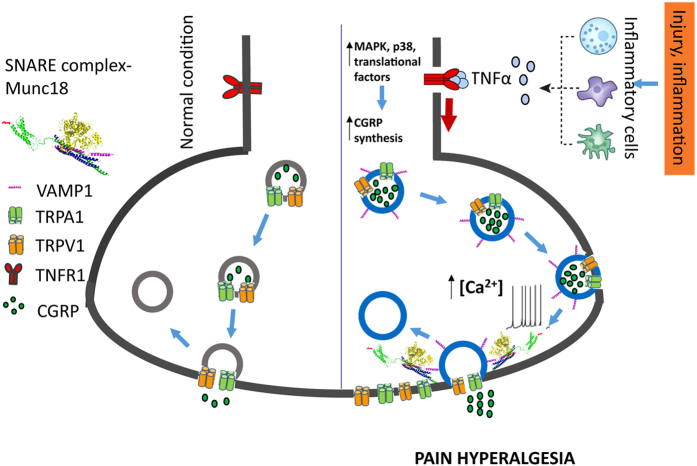
Schematic representation of potentiation by TNFα of exocytotic plasmalemma delivery of TRPA1 and TRPV1 channels and their co-trafficking. During injury and inflammation (right hand side), inflammatory cells release TNFα which binds to its receptor (TNFR) on sensory neurons. This results in activation of intracellular cascades, including MAPK, p38 and other translational factors to increase CGRP synthesis^72^. TNFα also elevates Ca^2+^ influx. These culminate in the enhancement of trafficking of CGRP-containing vesicles, and release of CGRP plus probably other mediators. In the case of sensory neurons expressing TRPA1 and TRPV1 channels, these proteins are packaged into CGRP- and VAMP1-containing vesicles and delivered to the plasma membrane involving the formation of SNARE complexes composed of SNAP-25, syntaxin 1, and VAMP1, as well as Munc18–1. It is reasonable to assume that equivalent processes apply in sub-populations of nociceptors that contain one or other of these TRP channels. Under such conditions, the delivery of these channels to the neuronal surface via such processes is elevated; this cascade likely contributes to the genesis of hyperalgesia and allodynia. In contrast, under normal conditions (left hand side) resting low levels of TRP channels are maintained by constitutive processes, apparently independent of BoNT-susceptible SNAREs. Our collective findings highlight the potential for ameliorating pain by targeting SNARE-protease inhibitors of neuropeptides exocytosis and surface delivery of TRP channels.

Elevated surface content of certain pain transducers in nociceptors is an important process underpinning chronic pain states^56^,^57^. During neurogenic inflammation, neuropeptides and pro-inflammatory cytokines act through a positive “feed-forward” loop, where the increased release of neuropeptides augments the secretion of cytokines; such amplification cascades culminate in hyper-sensitisation of nociceptors^58^. Hence, a pressing need existed for more information on a basis for inflammation-potentiated peripheral sensitisation–an undertaking fully warranted for both scientific and medical reasons. Herein, we tested the hypothesis that transportation of two TRP channels in cultured sensory neurons occurs in pain peptide-containing LDCVs, and this can be induced by an inflammatory cytokine, TNFα, via membrane fusion mediated by SNAREs (Fig. 8).

### TNFα-induced co-trafficking of TRPV1 and TRPA1 is a new phenomenon

Although TNFα is known to potentiate the capsaicin-sensitivity of spinal cord neurons^29^,^59^, it remained unclear whether or not this resulted from increased surface trafficking of the TRPV1 channel. Furthermore, the effect of TNFα on trafficking of TRPA1 channel was not known. Membrane insertion of TRPV1 and TRPA1 enhanced by TNFα was confirmed by the elevated labelling with their extracellular specific antibody, as well as the resultant functional consequences reflected by increased capsaicin- or AITC- evoked Ca^2+^ -influx. Interestingly, TRPV1 co-localised with TRPA1 on the fibres and cell bodies of cultured TGNs expressing both, and TNFα induced the surface trafficking for both channels to a similar extent. Strikingly, treatment with this cytokine elicited co-trafficking of TRPA1 and TRPV1, as confirmed by the PLA signals observed. In the current study, TNFα did not significantly change the total expression of TRPV1 measured by Western blotting (Fig. S6), in accord with an earlier finding on cultured synoviocytes^60^. Although it has been reported that TNFα can increase total TRPA1 expression in cultured primary DRG neurons, in a concentration dependent manner with a maximal effect observed at 15 ng/ml, 50 ng/ml only gave <20% increment in TGNs. Neither BoNT/A nor TNFα (100 ng/ml) changed the total expression of TRPA1 (Fig. S6). Thus, the enhanced surface expression of TRPV1 and TRPA1 is attributable to elevated transportation of vesicles containing these proteins, and their fusion with the plasmalemma rather than increased protein synthesis (Fig. 8).

Co-staining of TRPV1 and TRPA1 as well as various neuropeptides that reside in LDCVs, SgII, SV2 or VAMP-1 confirmed that both channels largely co-localise with these markers. Visualisation of TNFR1 and TRPV1 with their antibodies (Fig. S7) established that a majority of TGNs possess both, consistent with the observed effect of TNFα on the surface delivery of TRP channels. The observed ~3 fold increment of surface expression of TRPV1 and TRPA1 results from a prolonged effect of TNFα as quantitation of the CGRP content in the supernatant from TGNs that had been incubated for just 30 min with TNFα gave just ~8% increment observed herein. Obviously, this stimulant is quite different from depolarisation which elicited an >8 -fold increase in CGRP release over the same period^49^.

Since TRPV1 and TRPA1 occur in CGRP-releasing vesicles, and the intra-vesicular marker Syt-ecto highlights the exo-/endo-cytosis sites in sensory neurons^43^, it is not surprising that depolarisation unveiled a large extent of overlap in the signals for extracellular antibodies reactive with Syt-ecto and TRPV1 or TRPA1. Our findings indicate that fusion of neuropeptide-containing vesicles in TGNs is closely linked to the trafficking of nociceptive TRP channels, in accord with observations on DRG neurons^56^,^61^. Thus, it is possible that the inflammation-associated hyperalgesia produces not only an excessive release of pain mediators but, also, causes abnormally high levels of synaptic activity and sensitivity to pain, by up-regulating the surface availability of these transducing channels (Fig. 8). Such regulation of nociceptive proteins by chronic inflammation most likely contributes to the genesis and continuation of hyperalgesia and allodynia in disease conditions. Most recently, Tmem100, an inflammatory pain-related transmembrane protein, was found to co-express and complex with TRPA1 plus TRPV1 in DRG neurons, and potentiate TRPA1 activity in a TRPV1-dependent manner^62^. In our study, TNFα enhanced the co-trafficking of TRPA1 and TRPV1 in TGNs; thus, different mechanism(s) must be involved.

### SNAREs and Munc18 contribute to the membrane insertion of TRPV1 and TRPA1 in TGNs: BoNTs inhibit TNFα-evoked trafficking of these channels

We demonstrated that SNAP-25 and VAMP1 are important for the transportation of two TRP channels to the plasma membrane. Direct evidence for syntaxin 1 being essential could not be obtained from its cleavage by BoNT/C1 because it additionally truncates SNAP-25 (c.f. Fig. S2). Nevertheless, inhibition by Munc18–1 knockdown supports the involvement of all three SNAREs because Munc18–1 is known to be essential for facilitating the participation of syntaxin 1 in the formation of stable SNARE complexes that are required for synaptic exocytosis^63^,^64^,^65^,^66^. Munc18–1 allows the formation of a complex between syntaxin and SNAP-25 to serve as an acceptor for vesicle-bound VAMP and, thus, represents an intermediate in the exocytosis pathway^67^. Reduction of K^+^ -evoked CGRP release by BoNT/A or /D, respectively, correlates with their inhibition of TNFα-stimulated fusion of TRPV1 and TRPA1 with the plasma membrane^36^,^49^; knockdown of Munc18–1 yielded similar outcomes. Current data accords with an earlier observation that BoNT/A reduces trafficking of TRPV1 to the surface in *Xenopus* oocytes, and peptide mimetics that interfere with the function of SNAP-25 in membrane fusion block NGF-induced TRPV1 trafficking^19^.

### Normalization of excessive channel trafficking and transmitter release by BoNTs supports their potential as safe therapeutics to treat chronic neurogenic/inflammatory disorders

Interestingly, BoNT/A inhibits the increased Ca^2+^ -influx mediated by TRPV1 or TRPA1 in response to TNFα without lowering the normal level (Fig. 5) and reduces the augmented TRPV1 and TRPA1 surface delivery by TNFα. It is reasonable to conclude that during stimulation with TNFα, these channels are surface delivered via pain peptide-containing vesicles, and fuse at the exocytosis sites with simultaneous exposure of luminal domains of SV2 and synaptotagmin, the protein acceptors for several BoNTs (reviewed in^68^). Consequently, this resultant increased surface content of acceptors would facilitate the binding of BoNTs to these vesicle-releasing neurons. This, in turn, would augment their internalization with an eventual outcome of normalizing both transportation of the transducing channels and depolarisation-evoked transmitter release. In the clinic, BoNT/A successfully treats the refractory pain in patients suffering from persistent joint inflammation which previously proved unresponsive to anti-TNFα therapy and multiple intra-articular injections of corticosteroid^69^. A major advantage of the local BoNT/A therapy is that its benefit lasts 15–18 months in patients’ painful joints^69^. Furthermore, an engineered BoNT with extended action inhibits secretion of mucin and IL-8 induced by TNFα^70^. Thus, BoNTs and their derivatives offer encouraging prospects for widespread clinical applications in treating complex inflammatory and painful conditions.

In conclusion, it is shown for the first time that TNFα can induce the co-trafficking of TRPV1 and TRPA1 in close proximity via LDCVs in sensory neurons expressing both, and their trafficking requires at least 2 and probably all 3 SNAREs plus Munc18–1. Our findings are consistent with these channels being transported in the neuropeptide-containing LDCVs which allow them to function in pain and inflammation, contributing to the exacerbation and maintenance of chronic disease symptoms.

## Materials and Methods

### Materials

Cell culture materials, capsaicin, AITC, TNFα, β-tubulin III antibody and PLA reagents were bought from Sigma-Aldrich (Arklow, Co. Wicklow, Ireland). NGF-7S, Fluo-4 AM and anti-fade solution that includes DAPI were purchased from Bio-Sciences (Dun Laoghaire, Co. Dublin). Alomone Lab (Jerusalem, Israel) supplied rabbit polyclonal antibodies reactive with extracellular epitopes of TRPV1 or TRPA1 (ACC-029, ACC-037) and with total TRPV1 (ACC-030). SV2 monoclonal antibody was obtained from Developmental Studies Hybridoma Bank at University of Iowa. SP monoclonal and Munc18–1 antibodies were purchased from Abcam (Cambridge, UK). Goat anti-SgII was obtained from Santa Cruz Biotechnology (Santa Cruz, CA). Rabbit anti-VAMP1, 2 and 3 and monoclonal antibody for synaptotagmin I lumenal domain (Syt-ecto Oyster550, clone 604.2) were bought from Synaptic Systems GmbH (Goettingen, Germany). Monoclonal antibodies specific for CGRP or V5 were obtained, respectively, from Sigma-Aldrich and Thermo Fisher Scientific Inc. (Waltham, MA). SPI-BIO (Montigny Le Bretonneux, France) supplied the enzyme immuno-assay kit for CGRP. Donkey anti-rabbit Alexa-488, donkey anti-mouse or anti-goat Alexa-594 were supplied by Jackson ImmunoResearch (Hamburg, Germany). Ibidi GmbH (Martinsried, Germany) provided the culture chambers. Production of recombinant BoNT/A, /B, /C1 and /D and determination of their biological activities have been described^36^.

### Animals and ethics statement

Pups from rats (Sprague Dawley) bred in an approved Bio-Resource Unit at Dublin City University were used. The experiments, maintenance and care of the rodents complied with the European Communities (Amendment of Cruelty to Animals Act 1876) Regulations 2002 and 2005. Experimental procedures had been approved by the Research Ethics Committee of Dublin City University and licenced by the Irish authorities.

### Construction of plasmids

A lentiviral vector containing two human synapsin (hSYN) promoter and woodchuck hepatitis virus post-transcriptional regulatory element (WPRE) cassettes were obtained from Francisco Gomez Scholl^44^ and utilised to construct various DsRed- or GFP-fused genes. Rat TRPA1, synthetic VAMP1 and VAMP2 genes optimised for mammalian expression were individually inserted into the lentiviral vector between Sma I and Age I sites, in-frame with the GFP coding sequence; this generated constructs encoding TRPA1-GFP, VAMP1-GFP or VAMP2-GFP, respectively. DsRed gene was PCR amplified and inserted into the Not I and EcoRI site, with removal of the WPRE-SYN promotor-GFP cassette. The resultant plasmid was utilised to construct DsRed fusion genes. Briefly, rat TRPV1 and TRPA1 genes were PCR amplified and sub-cloned into the Bam HI and Not I sites, in-frame with DsRed coding sequence, to produce plasmids encoding TRPV1-DsRed and TRPA1-DsRed, respectively. Replacing DsRed gene with a short nucleotide sequence encoding a small peptide (GPAAAGKPIPNPLLGLDST; V5 tag, underlined) generated a construct encoding TRPV1-V5. Similarly, insertion of VAMP1 gene into the DsRed plasmid between Bam HI and Not I sites created a construct encoding VAMP1-DsRed fusion protein. For knock-down of Munc18–1, a pair of primers were synthesized with the following sequence: forward primer 5′ CCGGAAGGCACAGATGAAGAATCCCCTCGAGGGGATTCTTCATCTGTGCCTTTTTTTG-3′ and reverse primer 5′ AATTCAAAAAAAGGCACAGATGAAGAATCCCCTCGAGGGGATTCTTCATCTGTGCCTT-3’. Annealed primers were cloned into pLK0.1 puro vector between EcoRI and AgeI sites. All inserts in the constructed plasmids were confirmed by DNA sequencing. Production of lentiviral particles were performed using an established protocol^71^.

### Culturing of TGNs and lentiviral transfection

Dissociated trigeminal ganglia were dissected from postnatal day 3–5 Sprague Dawley rats, as previously described^49^, and kept in cold DMEM before digestion with collagenase I (1 mg/ml for 30 min at 37 °C). After washing and trituration, the dissociated cells were suspended in DMEM supplemented with 10% foetal bovine serum (BSA), 1% penicillin-streptomycin, and 100 ng/ml NGF-7S. High-density cultures (~50,000 neurons/well) were plated on poly-L-lysine and laminin double coated μ-slide 8-well Ibidi chambers or 35 mm μ-dish. After 7 days, the neuronal cultures were infected with lentiviruses harbouring TRPV1-DsRed, TRPA1-DsRed, TRPV1-V5, TRPA1-GFP, VAMP1-DsRed, VAMP1-GFP, VAMP2-GFP, GFP, DsRed or lentiviral shRNA targeting Munc18–1; its non-targeted version served as a control. The cells were then further incubated for 7–8 days before experimentation and imaging at day 14–15. In the case of BoNT treatment, TGNs infected with or without lentivirus expressing TRPV1-DsRed were incubated with 100 nM BoNT/A, /B, /C1 or /D at 37 °C for 24 h, then with TNFα (100 ng/ml) for the same period before processing for antibody surface labelling, Western blotting, Ca^2+^ imaging or PLA.

### Quantification of CGRP release

For the TGNs cultured on μ-Slide 8-well Ibidi chambers, medium was gently aspirated, 0.2 ml of basal release buffer (BR-HBS, mM; 22.5 HEPES, 135 NaCl, 3.5 KCl, 1 MgCl_2_, 2.5 CaC_l2_, 3.3 glucose and 0.1% BSA, pH 7.4) was added into each well, followed by a 30-min incubation at 37 °C. CGRP release stimulated with 1 μM capsaicin in BR-HBS was subsequently measured. To determine the amounts of CGRP released, 0.1 ml aliquots were added to 96-well plates coated with a monoclonal antibody against CGRP, and enzyme immuno-assay was performed following instructions for the kit. The basal release values obtained for each well were subtracted from those from capsaicin incubation to yield the evoked component.

### Live cell labelling and immunocytochemistry

Surface labelling of TRP channels followed a published protocol^18^. Briefly, live TGNs treated with or without TNFα were incubated with rabbit TRPV1-ecto (1:50) or TRPA1-ecto (1:25) well-characterized antibodies in culture medium for 10 min at 37 °C. After 5 rounds of washing with pre-warmed plain medium, the cells were incubated with donkey anti-rabbit Alexa Fluor 488 (1:2000) for 10 min at room temperature. Following removal of the secondary antibody by washing, the cells were fixed with 3.7% paraformaldehyde in Hank’s balanced salt solution (HBSS) for 30 min at room temperature. Specimens were mounted using anti-fade solution that included DAPI.

For conventional immuno-cytochemistry, TGNs on Ibidi culture chambers (μ-Slide 8-well) were fixed as above before permeabilisation in HBSS containing 0.1% Triton X-100. Samples were then blocked by 1% BSA, incubated with primary antibodies for 24 h at 4 °C before washing and applying fluorescent secondary antibodies for 1h at room temperature. The samples were mounted in anti-fade solution.

### Image acquisition and data analysis

TRPV1/A1 labelling experiments in live TGNs was imaged by a Zeiss LSM710 confocal microscope (Carl Zeiss MicroImaging) with argon and helium/neon lasers, using a 63 × 1.4 NA PlanAPO oil-immersion objective with the pinhole diameter set for 1 airy unit. Images acquired by Zen software (Universal Imaging, Göttingen) for all experimental groups were taken using identical acquisition parameters. Same settings were reused for different preparation of cultured neurons. For each experimental group, at least 5 cells were randomly chosen and analyzed. The mean fluorescence intensity in arbitrary units (a.u.) of each cell was determined by drawing aROI carefully encircling the surface of every cell body with the selection closed poly line. Background fluorescence for each cell was obtained by drawing a ROI area outside the cell surface. Mean background fluorescence intensity was then subtracted from the mean surface fluorescence intensity; the resultant values were pooled to give the means ± S.E.M at each condition.

### *In situ* PLA

Rat cultured TGNs, infected with lentivirus expressing TRPV1-V5 for 7 days, were treated with or without 100 nM BoNT/A for 24 h before incubation with TNFα (100 ng/ml) for 24 h in the presence or absence of BoNT/A. Cells were then fixed, permeabilised, blocked with 1% BSA for 1 h at room temperature and incubated overnight at 4 °C with monoclonal antibody against V5 (1:100) and rabbit polyclonal TRPA1 antibody (1:100). PLA was carried out according to the manufacturer’s instructions. Red fluorescent spots represent positive signals; negative controls were performed, likewise, except for omitting one or other of the primary antibodies. The red fluorescent spots per 10 μm^2^ of neurites in ROI randomly selected were counted and mean values plotted.

### Ca^2+^ influx, acquisition of confocal images and data analysis

TGNs grown in Ibidi-Dish 35 mm were loaded with 3 μM Fluo-4 AM in BR-HBS basal release buffer^49^ using a perfusion insert (Warner Instruments) at room temperature for 20 min, and placed on the Zeiss confocal microscope. An argon laser was used to excite the fluorophore at 488 nm. After ~2 min incubation in BR-HBS buffer, the baseline fluorescence was recorded for ~10 s. Then, the superfusate was switched to stimulation buffer containing 1 μM capsaicin or 50 μM AITC, and a timed series of images were continually taken for over 200s. Images of fluorescent signals were grabbed every ~3 s. The intensity of fluorescence at 505–530 nm (f) was analyzed offline on a cell-by-cell basis and expressed relative to the baseline fluorescence (f_0_) measured for each cell body in BR-HBS. Means ± SEM were plotted against time; n values are given in the figure legends.

### Statistical analysis

Data were presented as means ± S.E.M., probability values were determined with the use of Student’s 2-tailed *t* test, one-way ANOVA or two-way ANOVA followed by Bonferroni post hoc test by GraphPad Prism software, as specified in figure legends. *P* values <0.05 were considered significant.

## Additional Information

**How to cite this article**: Meng, J. *et al.* TNFα induces co-trafficking of TRPV1/TRPA1 in VAMP1-containing vesicles to the plasmalemma via Munc18-1/syntaxin1/SNAP-25 mediated fusion. *Sci. Rep.* **6**, 21226; doi: 10.1038/srep21226 (2016).

## Supplementary Material

Supplementary Information
