# Supplementary material for: TNFα induces co-trafficking of TRPV1/TRPA1 in VAMP1-containing vesicles to the plasmalemma via Munc18–1/syntaxin1/SNAP-25 mediated fusion
Source: Sci Rep. 2016 Feb 18;6:21226. doi: 10.1038/srep21226 (PMC4758037; doi:10.1038/srep21226)
Supplement: Supplementary Information [file srep21226-s1.pdf]

TNFα induces co-trafficking of TRPV1/TRPA1 in VAMP1-containing vesicles to the plasmlemma via Munc18-1/syntaxin1/SNAP-25 mediated fusion

Meng J, Wang J, Martin Steinhoff, J. Oliver Dolly

Supplementary Figures S1-S7

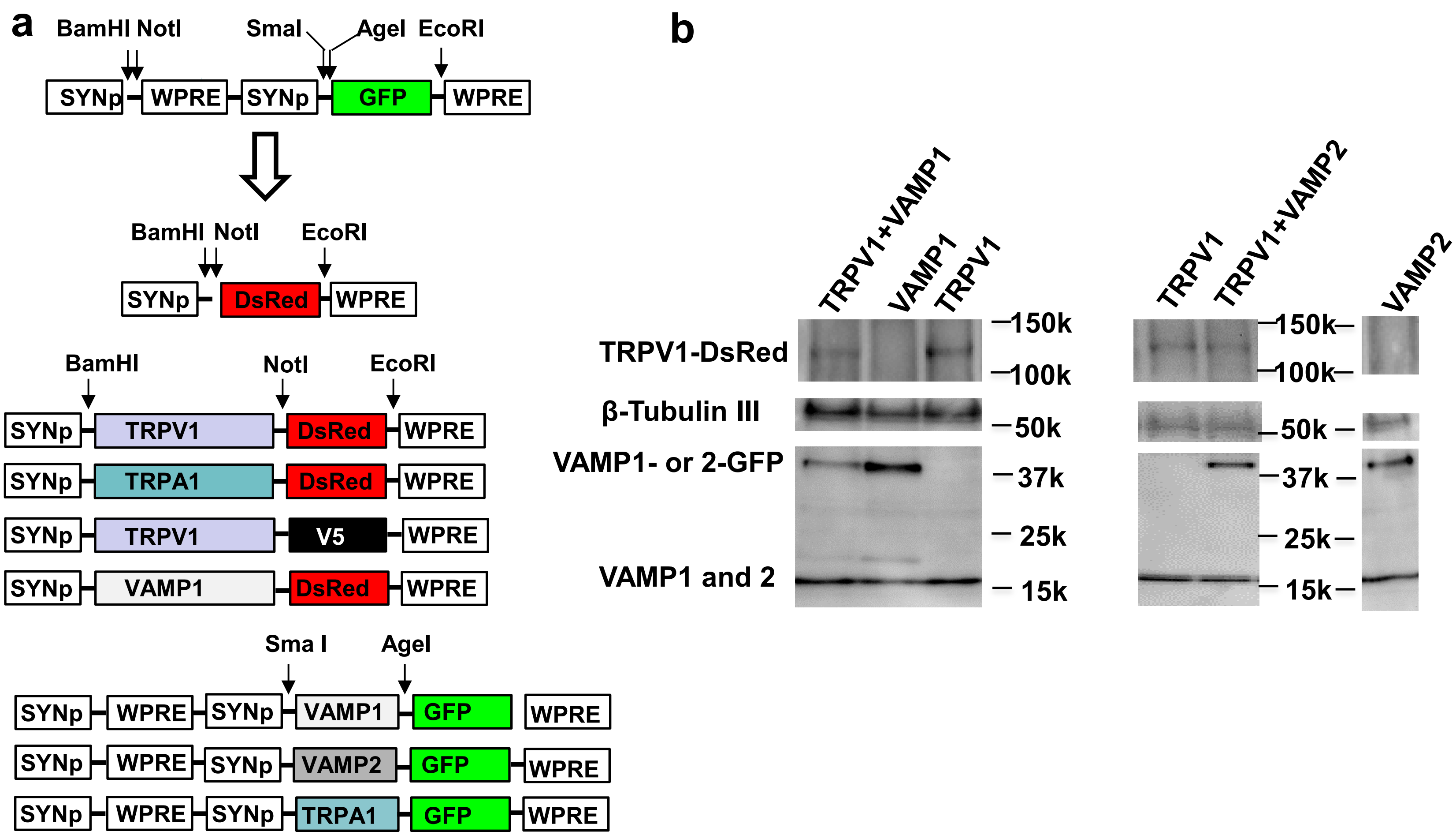

Fig. S1. Construction of lentiviral vectors and expression of TRPV1-DsRed, VAMP1-GFP and VAMP2-GFP by lentiviral-mediated gene-delivery. (a) Schematic of 8 lenti-viral vectors generated that express various proteins. Detailed cloning procedures are described in Materials and Methods. **(b)** Rat TGNs at 7 days in culture were infected by lentivirus expressing VAMP1-GFP, VAMP2-GFP or TRPV1-DsRed and, in some cases, TRPV1-DsRed with either VAMP1-GFP or VAMP2-GFP. The cells were then further cultured for 7-8 days before harvesting for probing the expression of these proteins by Western blotting. Exogenous TRPV1-DsRed fusion protein (~125 kDa) was detected in TGNs infected by lentiviral TRPV1-DsRed or co-infected with VAMP1- or VAMP2-GFP fusion viruses. Endogenous VAMP1 and VAMP2 (~17kDa) plus lentivirus expressed VAMP1-GFP and VAMP2-GFP fusion proteins (~ 44 kDa) were visualised using mixed VAMP1 and VAMP2 antibodies. β-Tubulin III serves as the internal control.

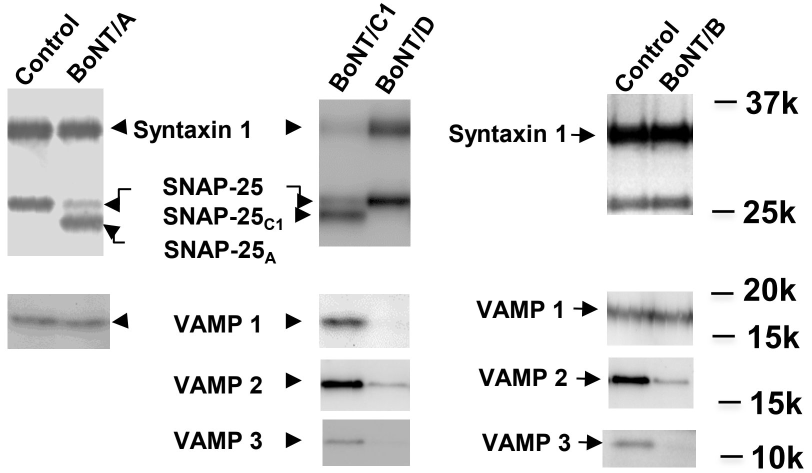

Fig. S2. Intracellular cleavage by BoNTs of their respective SNARE substrates. Rat cultured TGNs were treated with 100 nM BoNT/A, /B, /C1 or /D for 2 days before the cell lysates were harvested for SDS-PAGE and Western blotting. Immunoblots showing the patterns of cleavage of SNAP-25 by BoNT/A and /C1, which removed 9 and 8 residues from C-terminal of SNAP-25, respectively. BoNT/C1 additionally cleaved syntaxin 1. VAMP1, 2 and 3 were separately detected by isoform-specific antibodies. BoNT/B cleaved VAMP2 and 3 but not 1, unlike /D which truncated all three isoforms.

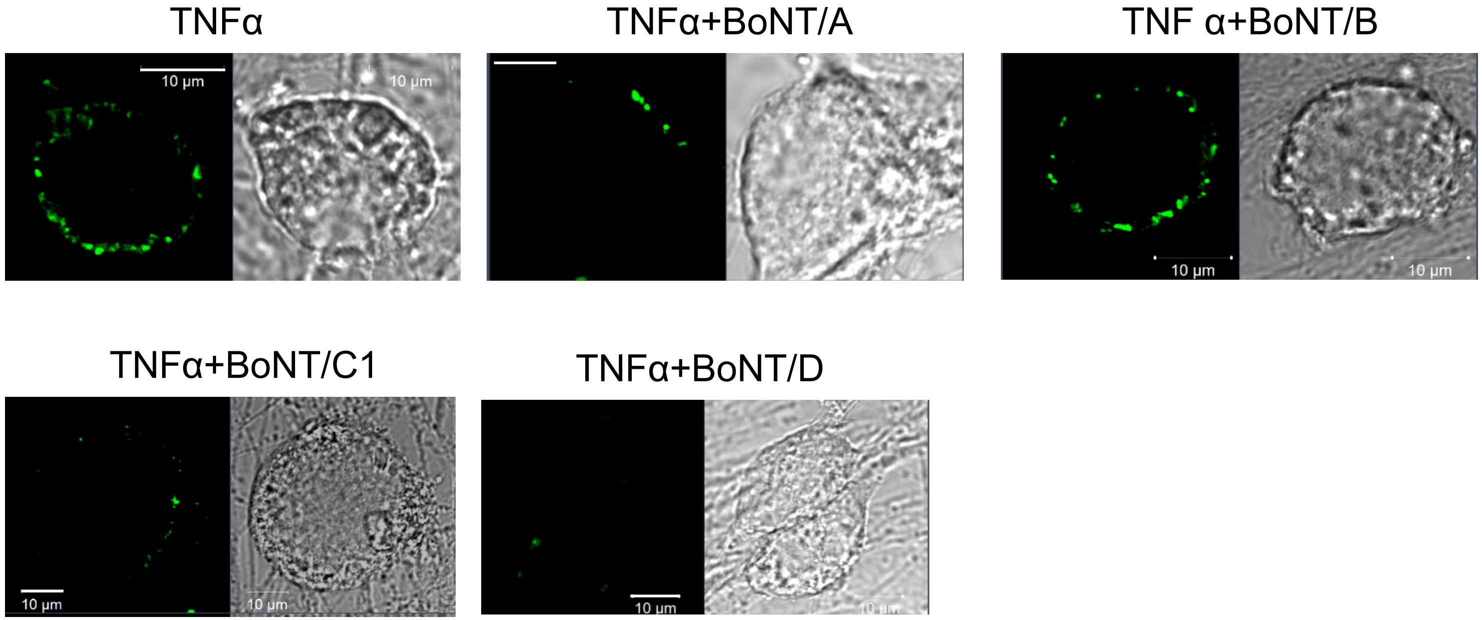

Fig. S3. Surface trafficking of TRPA1 in rat TGNs is blocked by BoNT/A or /C1 and /D but not /B. Cultured TGNs were pre-treated with 100 nM BoNT/A, /B, /C1 or /D for 24h before adding TNFα for 24 h and subsequent incubation for 10 min with TNFα, prior to TRPV1-ecto for surface labelling. It is apparent from these representative images that TNFα enhanced the surface trafficking of TRPA1 and this was reduced by pre-treatment with BoNT/A, /C1 or /D but not /B. Scale bars are 10μm.

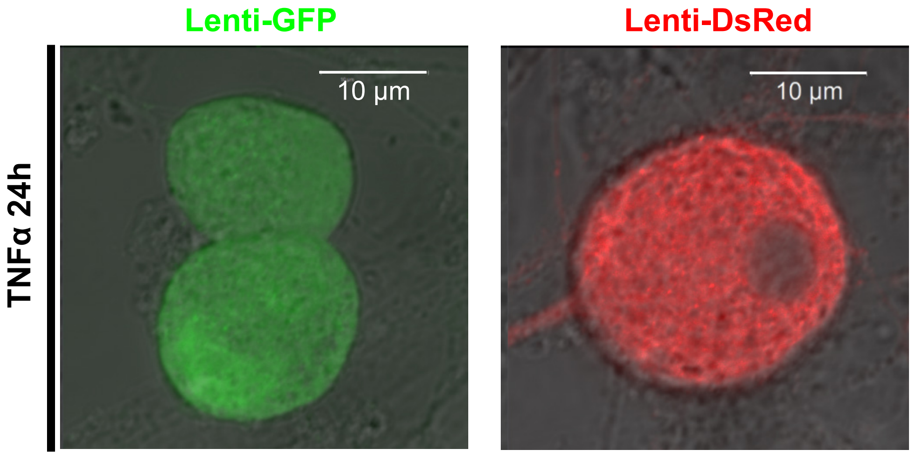

Fig. S4. Lentiviral-mediated expression of GFP and DsRed reporter proteins. TGNs at 7 DIV were infected by lentiviral GFP or DsRed and further cultured for 7 days. Then, these cells were treated with TNFα (100 ng/ml) in medium for 24 h before fixation and visualization by confocal microscopy. Lentiviral-expressed GFP and DsRed showed a diffuse pattern throughout the cytoplasm.

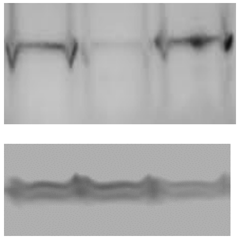

Fig. S5. Western blot analysis of the knockdown of Munc18-1 expression by shRNA . TGNs expressing TRPV1-DsRed were infected with lentiviral-shRNA selectively targeting Munc18-1, or its non-targeted control, for 7 days before harvesting, and Western blotting using antibody against Munc18-1. Munc18-1 expression was reduced by its specific shRNA. β-Tubulin III was probed as a loading control.

a

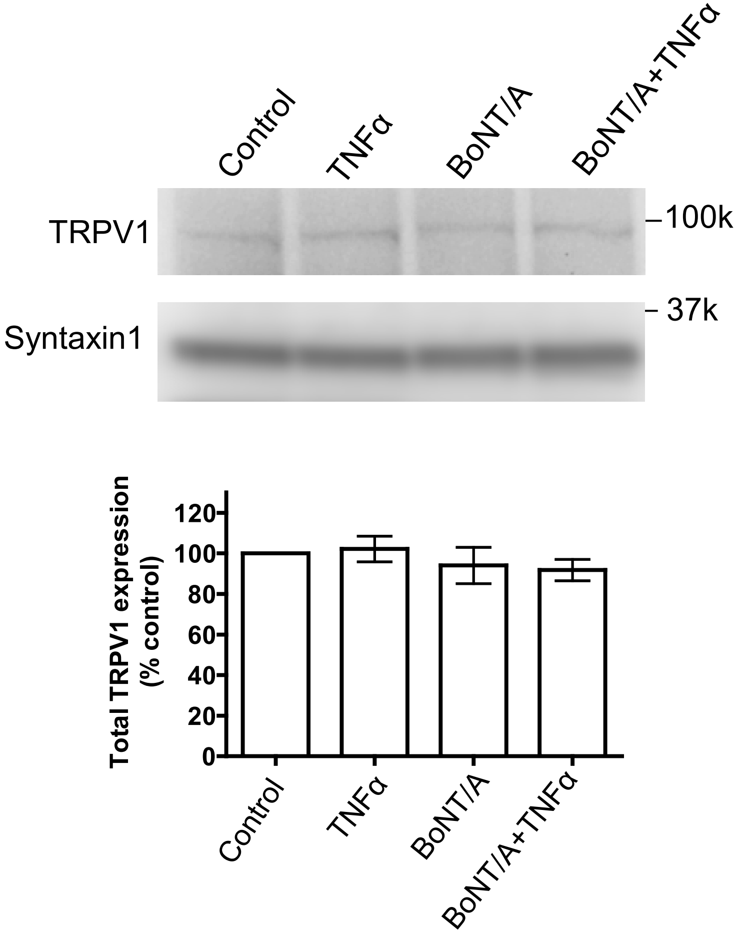

b

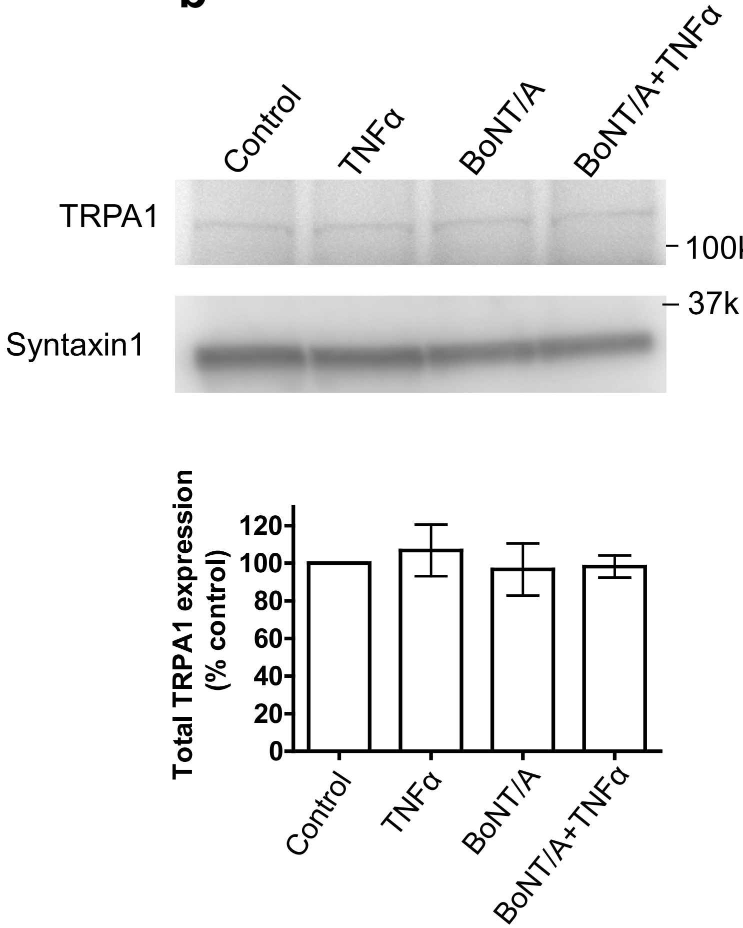

Fig. S6. TNFα and BoNT/A did not significantly alter the total expression of TRPV1 and TRPA1. Rat TGNs at 7 DIV were treated with or without 100 nM BoNT/A for 24h before incubation with or without 100 ng/ml TNFα for another 24h. Samples were harvested in LDS sample buffer and subjected to SDS-PAGE followed by Western blotting, using antibodies against TRPV1 (a) or TRPA1 (b). Syntaxin 1 was probed as internal loading control. The expression level of TRPV1 and TRPA1 were quantified relative to syntaxin 1 and calculated as % of non-treated control. Data plotted are the means±S.E.M. from 3 independent experiments. Note that there is no significant difference between each group. One-way ANOVA paired values followed by Bonferroni post hoc test was used.

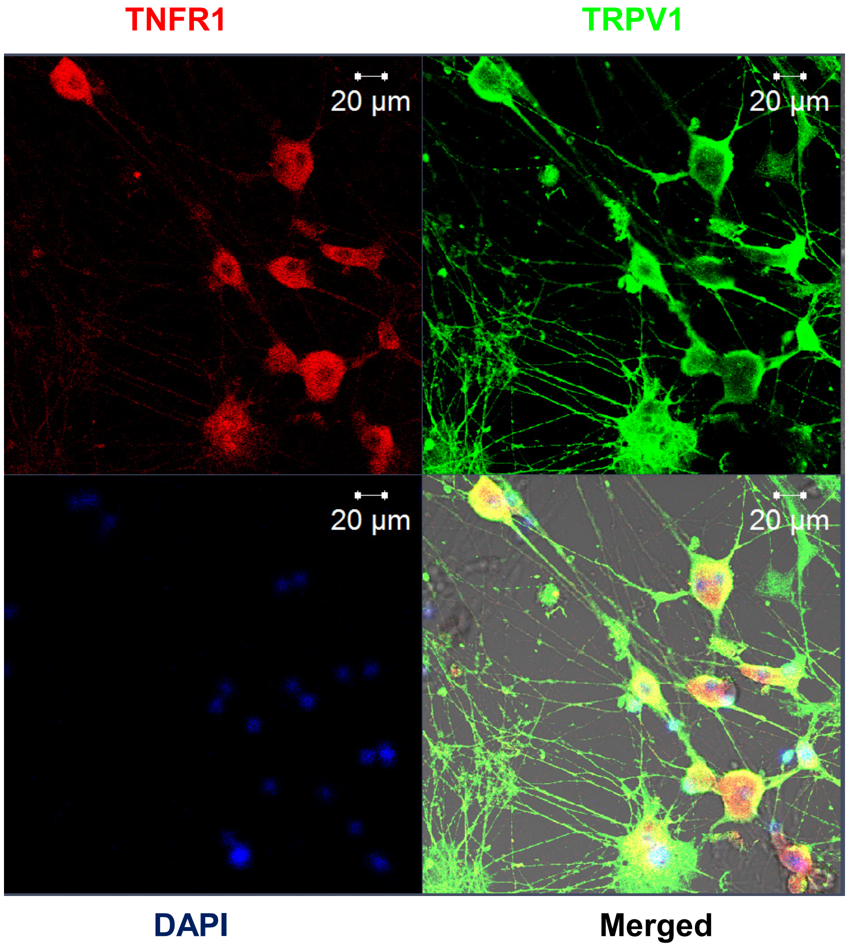

Fig. S7. A majority of cultured TGNs express both TNFR1 and TRPV1. Rat TGNs were fixed, permeabilized and blocked with 1% BSA before staining with mouse antibody against TNFR1 and rabbit TRPV1 antibody. After extensive washing, cells were labelled with fluorescent secondary antibodies and counter-stained with DAPI before the images were taken using a confocal microscope, in fluorescence and phase-contrast modes.
